# The effects of upper versus lower extremity resistance training in patients with breast cancer: a systematic review

**DOI:** 10.2340/1651-226X.2025.44387

**Published:** 2025-11-19

**Authors:** Hacer Oncu, Ceyhun Topcuoglu, Ebru Calik, Melda Saglam, Naciye Vardar Yagli

**Affiliations:** Department of Cardiorespiratory Physiotherapy and Rehabilitation, Faculty of Physical Therapy and Rehabilitation, Hacettepe University, Ankara, Turkey

**Keywords:** Breast cancer, resistance training, upper extremity, lower extremity, quality of life, muscle strength, exercise capacity, adverse effect

## Abstract

**Purpose:**

The aim of this systematic review was to analyze the effects of upper, lower and upper-lower extremity combined resistance training (RT) in breast cancer survivors.

**Methods:**

A systematic literature search was performed using ClinicalTrials.gov, Cochrane Library, PubMed, Scopus, EBSCO, and Web of Science databases. Randomized controlled trials published between 1970 and April 30, 2025 comparing upper extremity RT, lower extremity RT and combined upper and lower extremity RT; comparing upper extremity RT and/or lower extremity RT in the experimental group and no RT in the control group or the sham group in the control group were examined.

**Results:**

We included 16 studies with 1,207 participants. Upper extremity RT training programs reduce shoulder pain, arm disability and improve upper limb muscle strength. Lower extremity RT training programs improve maximum strength, level of physical activity (PA), 6-minute walking test (6MWT) distance, muscle fatigue indicators and maximal voluntary isometric contraction values. Combined upper-lower extremity RT trainings enhance 6MWT distance, muscular strength, walking speed, body image, and reaction time.

**Interpretation:**

Upper, lower, and combined upper-lower extremity RT programs can be beneficial rehabilitation therapies for breast cancer survivors. As the effects of the type of RT are different from each other, the specific needs of each patient should be considered while designing the ideal RT in breast cancer survivors.

## Introduction

Breast cancer has replaced lung cancer as the most commonly diagnosed cancer worldwide, now accounting for 1 in 8 cancer diagnoses and 2.3 million new cases in both sexes [1, 2]. Almost 85–90% of breast cancer cases occur sporadically due to environmental and age-related causes and 10–15% are inherited, especially from mutations in the BRCA1 and BRCA2 genes [3]. Increased survival rates after advances in treatments have led to more complications related to breast cancer and its treatments [4, 5]. The most common complications related to breast cancer are pain in the upper extremities, limitation in joint movements, functional limitation, decreased exercise capacity, breast cancer-related lymphedema (BCRL), fatigue, stiffness, risk of infection, loss of sensation; and these complications may occur in the short or long term. These adverse effects also lead to a decrease in quality of life and functional capacity [6]. Exercise and physical activity (PA) have been shown to reduce the risks of breast cancer development, mortality and recurrence [7, 8]. In addition to the positive effects of exercise on physical, psychological, cognitive, and social well-being, studies have comprehensively demonstrated its role in preventing many chronic diseases and improving quality of life and functional recovery [9]. Breast cancer survivors may sometimes avoid exercising due to physical symptoms and psychological barriers and they prefer pain-free activities such as walking [10]. However, evidence indicates that this assumption is incorrect [11, 12]. It has also been reported to be effective in reducing adverse effects such as loss of joint mobility and muscle strength, pain, fatigue, anxiety and depression associated with breast cancer and its treatments [13–15]. Exercise training studies mostly consist of aerobic training (AT) or resistance training (RT) combined with AT in breast cancer. RT modality used as a stand-alone intervention is less frequently implemented than other types of training [11]. In fact, it has been found that RT improves muscle strength, range of motion (ROM), quality of life, functional level and reduces negative effects of disease and treatments such as depression and fatigue [16]. A systematic review on RT in breast cancer reported that RT is typically performed twice a week on strength training machines, using a load between 50 and 80% of one-repetition maximum (1RM), in 60-min sessions, with two or three sets of 8 to 12 repetitions for each muscle group worked [17]. RT is among the interventions that should be included in rehabilitation programs for patients with breast cancer accompanying BCRL. RT increases lymphatic drainage, thereby reducing the risk of lymphedema development, and also helps in reducing the volume of the affected limb besides improving muscle strength [17]. These concerns emphasise the need for a clearer understanding of the optimal exercise prescription particularly the intensity, duration and frequency of RT for preventing occurrence and progression of BCRL [18]. Existing studies in the literature suggest that RT progressing from low to high intensity increases lymphatic drainage, thus helping to reduce lymphoedema [18]. Studies have shown that high-intensity RT is more effective than low-intensity RT in reducing lymphedema and increasing muscle strength. Breast cancer patients with lower tolerance to high intensity exercise can maximise the benefit in managing lymphedema by appropriately increasing the frequency and duration of exercise for at least 12 weeks to ensure the positive effects [18]. However, there is still no consensus on the effectiveness of different RT methods and the optimal training mode for RT on BCRL in patients with breast cancer [19].

The number of studies comparing the effectiveness of different RT modalities in individuals with breast cancer is insufficient. Examining the effects of RT applied to different limbs in individuals with breast cancer and performing the most effective type of resistance exercises could contribute to reducing disease-related activity limitations, healthcare costs, and mortality. As a result, the aim of this systematic review was to compare the effects of upper limb, lower limb and combined upper-lower limb RT on muscle strength, quality of life, exercise capacity, and BCRL considered as an adverse effect in breast cancer. These findings may provide guidance for implementing most appropriate rehabilitation programs without any adverse events in individuals with breast cancer. In this way, different types of RT can be added to the rehabilitation programs of patients with breast cancer, meeting rehabilitation needs of patients.

## Materials and methods

**Figure 1:**
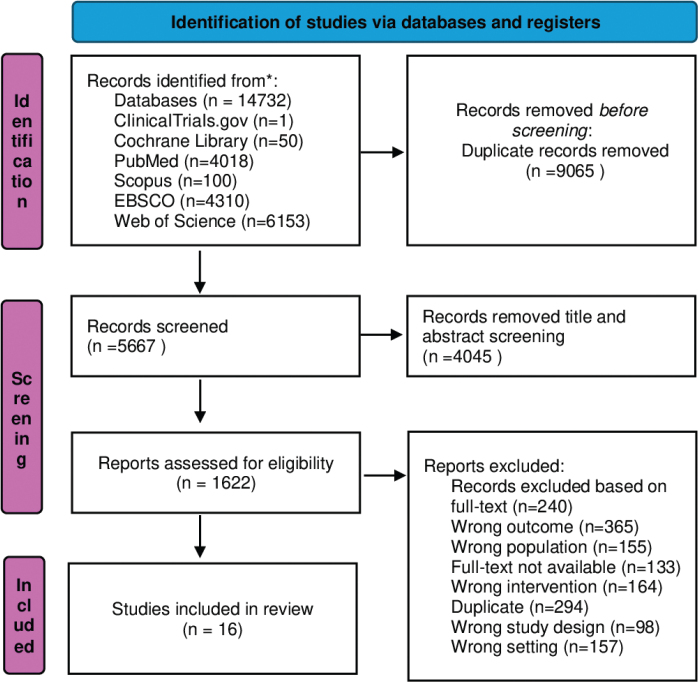
Preferred Reporting Items for Systematic Reviews and Meta-Analyses flowchart. Study identification and process for selection of studies included in the review [47].

We conducted this systematic review according to the Cochrane guidelines [20] and reported it according to the Preferred Reporting Items for Systematic Reviews and Meta-Analyses (PRISMA) 2020 Checklist (Figure 1) [21].

### Search strategy

The PICO model (Population, Intervention, Control and Outcome) was used in the selection of the articles. The study population included patients with breast cancer. Randomized controlled studies comparing upper extremity RT, lower extremity RT and combined upper and lower extremity RT; comparing upper extremity RT and/or lower extremity RT in the experimental group, and no RT in the control group or the sham group were examined. ClinicalTrials.gov, Cochrane Library, PubMed, Scopus, EBSCO, and Web of Science databases were used. Study titles and abstracts were independently reviewed for relevance by two authors (CT and HO) to identify studies that met inclusion and exclusion criteria, study design, sample characteristics, intervention programs, and the effects of exercise. When a disagreement arose, all authors negotiated until a consensus was reached. If only the abstract were available or data were missing, the authors of the publication were contacted. Two reviewers independently extracted data from the full text of the included studies and recorded details about study design, interventions, patients, and outcome measures in a standard predefined Microsoft Excel spreadsheet. When outcome data were collected at different time points in studies, data collected immediately after the completion of the intervention were included in the analyses.

### Inclusion and exclusion criteria

We included randomised controlled trials (RCTs) in this systematic review. Non-randomised trials, prospective and retrospective cohort studies, cross-sectional studies, case–control studies, systematic reviews, case reports, letters, editorials, qualitative studies, conference abstracts, posters, and protocols were excluded. Preprints were not included, but verified if the corresponding articles had already been published. We included randomized controlled trials comparing upper extremity RT and/or lower extremity RT in the experimental group, no RT in the control group, or sham group; comparing upper extremity RT, lower extremity RT, or combined upper and lower extremity RT in the experimental group, no RT in the control group, or sham group. We included RT with elastic bands, elastic tube or elastic balls, free weights, and weight machines. Use of other training modalities (such as aerobic exercises, balance exercises) in combination with RT were excluded. Studies that only had abstracts (from conferences, study groups, etc.) and studies with recommendations without detailed interventions and no further details were excluded. There were no restrictions in terms of language, country, race, or gender.

### Selection and data extraction

Search results were imported into a reference management program (Endnote 20.2 (Bld 15.709), Clarivate Analytics). Based on inclusion and exclusion criteria, two reviewers independently screened all records by title and abstract. Subsequently, two reviewers independently reviewed the full text of all potentially relevant records, using the same selection criteria. Data were extracted by one reviewer (HO) and checked by two other reviewers (CT, MS) using standardised data extraction forms in Covidence and Microsoft Excel (Version 2202). Any queries or disagreements in either of the steps above were resolved through discussion or, if necessary, by consulting another reviewer. The following data items were extracted: title, authors, publication year, journal, publication status, country, timeframe of patient recruitment, study design, population characteristics (i.e. inclusion and exclusion criteria, setting, sample size, age, sex, follow-up time since breast cancer, breast cancer stage, applied breast cancer treatment), rehabilitation therapy, comparator(s), primary and secondary endpoints, and the main results. Data were sought for the following outcome measures: shoulder pain, arm disability, the thickness of the subcutaneous tissue, muscle strength (isotonic, isometric and isokinetic), body composition, BCRL, pain and the sensation of heaviness in the affected limb during activities of daily living (ADLs), quality of life, shoulder ROM, level of PA, 6-minute walking test (6MWT) distance, circulating inflammatory markers, walking speed (4m Walk), and muscular endurance (sit-up and plank test).

### Quality assessment

The Physiotherapy Evidence Database (PEDro) scale was used to assess the methodological quality of randomized controlled trials [22]. The PEDro scale consists of 11 methodological items. The first item is not scored and overall assesment is expressed over a total of 10 points. While scores below 4 are expressed as ‘poor’, scores between 4 and 5 are expressed as ‘moderate’, scores between 6 and 8 are expressed as ‘good’, and scores between 9 and 10 are expressed as ‘perfect’ [23]. The two authors independently scored the publications and disagreements were resolved by negotiating with a third party [24, 25].

### Risk of bias assessment

The Cochrane risk-of-bias tool was used to assess the risk of bias in the following studies included studies. This study assessed attrition bias, reporting bias, other bias methods. Risk of bias was assessed by two independent authors. In case of disagreement, the authors negotiated with a third party [26].

### Protocol registration

We published and prospectively registered the protocol for this systematic review on PROSPERO (CRD42023489534).

## Results

### Identification of studies

From the database search on 30 April 2025, 15,634 records were identified, of which 12,234 were screened for title and abstract. Of those, 321 references were screened based on full-text, of which 16 unique studies were retained for this review (Figure 1).

Eight studies including upper extremity RT, 3 studies including lower extremity RT and 5 studies including combined upper and lower extremity RT were evaluated. Characteristics of included studies are presented in Table 1.

**Table 1:** Characteristics of included studies.

Reference	Sample size^b^	Patient population	Measurement, intervention studied
Naczk et al. 2022 [27]	24 women	Survivors with breast cancer who have undergone partial or simple mastectomy	Shoulder muscles strength Body composition BRCL Inertial training 6 weeks, twice a week A warm-up and 4 sets of shoulder flexion, extension, abduction and adduction exercises (16 sets for each arm) for each session. Each set 15 seconds (2 minu breaks between sets) for 12–14 repetitions
Kilbreath et al. 2006 [28]	22 women	Survivors with breast cancer undergoing breast cancer treatment including axilla surgery	Quality of life BCRL Shoulder ROM Shoulder muscle strength 2 sets, 8 to 12 repetitions moderate resistance exercises (Shoulder flexors and abductors and external rotators) Free weights for the weekly supervised sessions A suitable Thera-band® rating for the home program 15 (difficult) target intensity on the Borg Effort Scale
Sagen et al. 2009 [31]	204 women	Survivors with breast cancer who have undergone axillary node dissection	BRCL Pain and sensation of heaviness in the affected limb Moderate RT program 2–3 times a week for 6 months RT (total 45 min) 15 repetitions for each exercise Low resistance (0.5 kg) during the first 2 weeks
Bok et al. 2016 [32]	32 women	Survivors with breast cancer who have BCRL	BRCL Subcutaneous thickness Muscle thickness Progressive resistance exercise (PRE), complex decongestive physiotherapy. Resistance exercises: (1) dumbbell fly, (2) triceps extension, (3) single arm bent-over row, (4) biceps curl, (5) dumbbell side raise and (6) forearm raise using 0.5 kg dumbbells 5 repetitions twice a day and progression by adding 5 more repetitions each week during 8 weeks
Kilbreath et al. 2011 [33]	160 women	Survivors with breast cancer undergoing breast cancer treatment including axilla surgery	Quality of life Shoulder ROM Shoulder strength BCRL Home program of passive stretching, PRT for shoulder muscles. 2 sets, 8 to 12 repetitions of moderate resistance exercises (Shoulder flexors and abductors and external rotators) Free weights for the weekly supervised sessions A suitable Thera-band® rating for the home program
Do et al. 2015 [29]	44 women	Survivors with breast cancer who have BCRL	Shoulder muscular strength DASH BCRL Quality of life Moderate intensity resistance exercise program using an elastic band (theraband) for 8 weeks Intensive complex decongestive physiotherapy for 1 or 2 weeks (depending on severity) The exercise intensity: > 6 on OMNI Scale 3 sets, 10 repetitions of isolated shoulder flexion, isolated shoulder abduction and extension, and internal rotation and external rotation 5 times a week for 8 weeks
Aboelnour et al. 2022 [30]	70 women	Breast cancer patients with unilateral post-mastectomy adhesive capsulitis	Shoulder range of motion Shoulder muscle strength DASH Pain The traditional physical therapy program, Thera-Band exercises for shoulder muscles and scapular stabilization exercises 5 days a week for 60 min during 8 weeks. 2–3 sets of 10–15 repetitions of each exercise for 30 min All patients started the strengthening exercises with the color yellow, progressing to red and then to green Progression was applied when the patient could easily complete 3 sets of 10–15 repetitions
Ibrahim et al. 2018 [34]	59 women	Survivors with young (aged 18–45 years) breast cancer	Shoulder muscle ROM Handgrip strength Pain with shoulder movements Upper limb strengthening, endurance and stretching exercises exercise with an exercise band 2 times a week for 12 weeks 8–10 RM exercise intensity
Santagnello et al. 2020 [35]	20 women	Survivors with breast cancer	Self-reported fatigue Body composition Physical performance Maximum strength Muscle power Resistance exercises (leg extension, leg curl, 45° leg press and calf raise) %80 of 1RM exercise intensity 8–12 repetitions for 12 weeks
Martins et al. 2023 [36]	22 women	Survivors with breast cancer	Self-reported fatigue Sitting time Level of PA Fat percentage Muscle fatigue indicators Maximal voluntary isometric contraction Six-minute walk test Blood sample Muscle strength RT (leg extension, leg curl, 45° leg press, and calf raise) 3 sets of 8–12 repetitions with 80% of 1RM 3 times a week for 12 weeks
Cešeiko et al. 2019 [37]	55 women	Survivors with breast cancer with disease stage I-III	Quality of life Exercise tolerance Muscle strength Fatigue RT (leg extension, leg curl, 45° leg press and calf raise) 80% of 1RM, 8 to 12 repetitions 3 days per week for 12 weeks Week 1: 1 set (4 exercises) of 15–20 repetitions at ~60% of 1RM; Week 2: 2 sets of 12–15 repetitions at ~70% of 1RM; Week 3–12: 3 sets of 8–12 reps at ~80% of 1RM
Winters-Stone et al. 2022 [38]	114 women	Survivors with breast cancer with early-stage, post-treatment, older	Physical function Self-report physical function 6 minute walk test Maximal muscle strength Flexibility or ROM Progressive low-moderate intensity exercise programs: aerobic, resistance or flexibility (active control) training. 12 months of supervised followed by 6 months of unsupervised exercise training 5 upper body and 5 lower body exercises: chair pose, lunges (front, back, side), calf raises, calf raises, single arm rows, chest press, forward/lateral shoulder raises and push-ups. 2–3 sets of each exercise with a maximum of 10–15 RM. Lower extremity exercises were performed with wearing a vest with a weight that was gradually increased or tolerated from 1 to 10% of the participant’s body weight
Schmitz et al. 2005 [39]	85 women	Survivors with breast cancer	Body size Biomarkers Maximal muscle strength Twice-weekly weight training
Schmitz et al. 2010 [41]	154 women	Survivors with breast cancer survivors 1 to 5 years postunilateral breast cancer, with at least 2 lymph nodes removed and without clinical signs of BCRL	BCRL Body fat Dietary intake Self-reported PA Maximal muscle strength Upper body exercises: Seated row, supine dumbbell press, side or front lift, biceps curl and triceps pushdown were performed with dumbbells or machines with variable resistance. Lower body exercises: Leg press, back extension, leg extension and leg curl were performed with resistance machines. 90 min RT twice a week for 13 weeks Three sets, 10 repetitions per set in each session After 13 weeks, participants continued unsupervised exercise twice a week for 1 year.
Speck et al. 2010 [40]	234 women (112 with lymphedema)	Survivors with breast cancer	Body image Upper and lower body strength Quality of life Strength training. Twice a week for 90-min sessions during the first 13 weeks. New weight-bearing exercises with little or no resistance during the first eight sessions. After two sessions, resistance was increased in minimal increments (1/2 pound) for each upper body exercise. Upper body exercises: Seated rows, supine dumbbell press, side or front raises, biceps curls and triceps pushdowns. Lower body exercises: Leg press, back extension, leg extension, and leg curls. Three sets, 10 repetitions per set in each session After 13 weeks, participants continued unsupervised exercise twice a week for up to 1 year.
Lee et al. 2022 [42]	30 women	Survivors with breast cancer	Body composition Muscle strength Muscular endurance Flexibility Reaction time Balance Inflammation Immune cell 12 weeks of high-intensity circuit resistance exercise (leg press, seated row, leg extension, shoulder press, back extension, arm extension, hip adduction and hip abduction exercises) The exercise set consisted of warm-up (walking and stretching, 10 min), main exercise (30 min) and cool-down (stretching, 10 min), each set was followed by a 3-min rest period. Pneumatic exercise equipment 2–3 times a week for 50 min a day for 12 weeks The exercise intensity was progressed as following: 16 repetitions, 3 sets of 1 RM 40% in the first week 12 repetitions, 4 sets of 1 RM 60% in the second week 12 repetitions, 4 sets of 1 RM 60% in the third week 12 repetitions, 4 sets of 1 RM 60% in the third week 8 repetitions, 4 sets of 1 RM 80% in a 1:1 concentric/eccentric ratio

1RM: one-repetition maximum; BCRL: breast cancer-related lymphedema.

### Risk of bias

**Figure 2:**
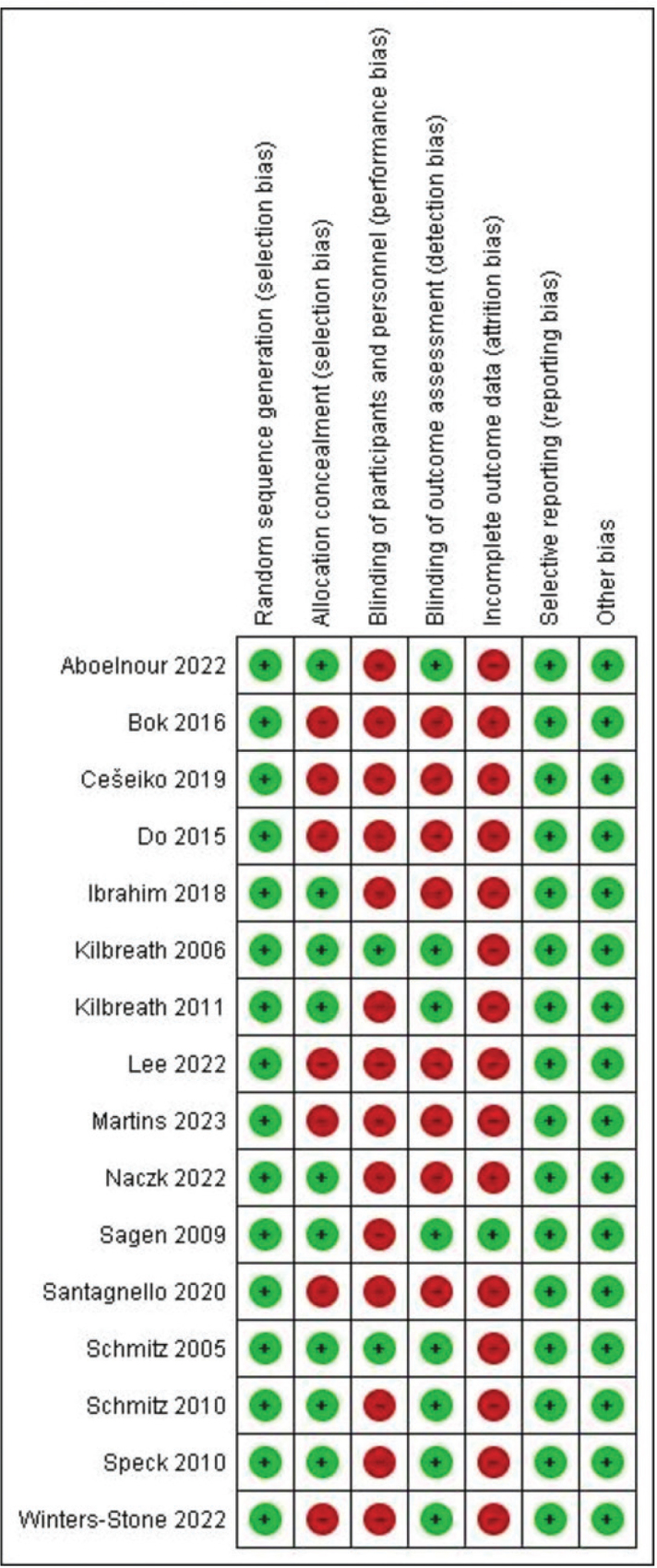
A summary of the risk of bias for the trials included in this systematic review.

Sixteen articles reported random sequence generation for selection bias [27-42], and nine of them had allocation concealment [27, 28, 30, 31, 33, 34, 39-41]. Only two studies blinded participants and physiotherapists [28, 39], while other studies had performance bias. For detection bias, blinding was reported in the outcome evaluation of eight articles [28, 30, 31, 33, 38-41]. In only one study, all participants completed RT [31], and all data were provided. In the remaining articles, the ‘intention to treat’ analysis was not given, and there was an attrition bias. There was no reporting bias or other bias in any of the studies (Figure 2).

### Effects of rehabilitation interventions

**Figure 3:**
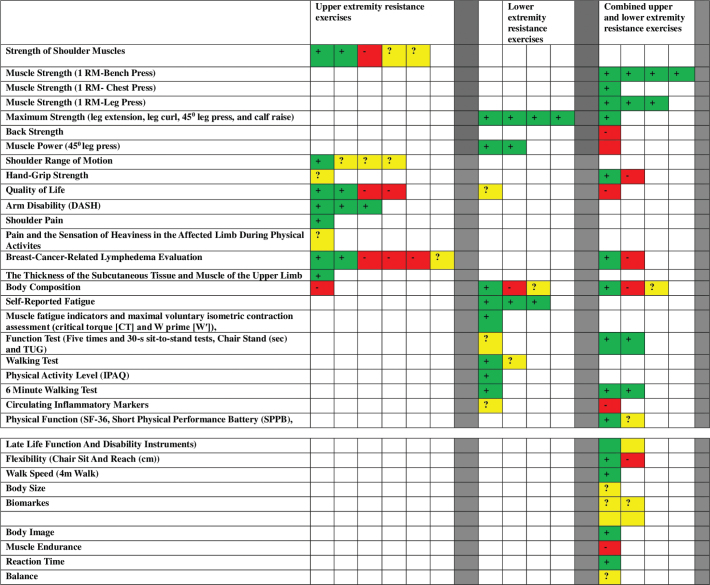
Graphical summary of the results of the included studies. + (green) = beneficial results, ? (yellow) = conflicting results, – (red) = no significant improvement.

Figure 3 graphically summarises the results of the included studies.

### Quality of studies

Quality assessment was conducted for each study using the PEDro score. The PEDro score of the included studies was between 4 and 9. Quality of one article was perfect, quality of six articles was good, and quality of nine articles was moderate (Table 2).

**Table 2:** Quality Assessment of Randomized Clinical Trials – PEDro Scale.

The Authors	Eligibility criteria	Random allocation	Concealed allocation	Baseline Comparability	Blinding (participant)	Blinding (therapist)	Blinding (assessor)	Adequate Follow-Up	Intention to Treat Analysis	Between-Group Comparisons	Point Estimates and Variability	Total
**Upper extremity resistance exercises**												
Naczk et al. 2022 [27]	Yes	Yes	Yes	Yes	No	No	No	No	No	Yes	Yes	5
Kilbreath et al. 2006 [28]	Yes	Yes	Yes	Yes	Yes	Yes	Yes	Yes	No	Yes	Yes	9
Sagen et al. 2009 [31]	No	Yes	Yes	Yes	Yes	No	Yes	No	Yes	Yes	Yes	7
Bok et al. 2016 [32]	Yes	Yes	No	Yes	No	No	No	No	No	Yes	Yes	4
Kilbreath et al. 2011 [33]	Yes	Yes	Yes	Yes	No	No	Yes	Yes	No	Yes	Yes	7
Do et al. 2015 [29]	Yes	Yes	No	Yes	No	No	No	Yes	No	Yes	Yes	5
Aboelnour et al. 2022 [30]	Yes	Yes	Yes	Yes	No	No	Yes	No	No	Yes	Yes	6
Ibrahim et al. 2018 [34]	Yes	Yes	Yes	Yes	No	No	No	No	No	Yes	Yes	5
**Lower extremity resistance exercises**												
Santagnello et al. 2020 [35]	Yes	Yes	No	Yes	No	No	No	No	No	Yes	Yes	4
Martins et al. 2023 [36]	Yes	Yes	No	Yes	No	No	No	No	No	Yes	Yes	4
Cešeiko et al. 2019 [37]	Yes	Yes	No	Yes	No	No	No	Yes	No	Yes	Yes	5
**Combined upper and lower extremity resistance exercises**												
Winters-Stone et al. 2022 [38]	Yes	Yes	No	Yes	No	No	Yes	No	No	Yes	Yes	5
Schmitz et al. 2005 [39]	Yes	Yes	Yes	Yes	Yes	Yes	Yes	No	No	Yes	Yes	8
Schmitz et al. 2010 [41]	Yes	Yes	Yes	Yes	No	No	Yes	Yes	No	Yes	Yes	7
Speck et al. 2010 [40]	Yes	Yes	Yes	Yes	No	No	Yes	No	No	Yes	Yes	6
Lee et al. 2022 [42]	Yes	Yes	No	Yes	No	No	No	Yes	No	Yes	Yes	5

### Upper extremity resistance exercises

Eight RCTs [27-34] have applied upper extremity RT in women after breast cancer. Included studies used inertial training [27]; 3 studies used elastic resistance bands [28-30]; 2 studies used free weights [31, 32]; one study used both free weights and elastic resistance bands [33]; and one study used resistance bands and instructions [34].

Two studies evaluating shoulder muscle strength reported positive results, while one study reported that no results could be obtained and two studies reported conflicting results. One study evaluating shoulder joint ROM reported positive results, while three studies reported conflicting results. One study evaluating handgrip strength has reported conflicting results. While two studies evaluating quality of life reported positive results, two studies reported no results. Three studies evaluating arm disability by the DASH score reported positive results. One study evaluating shoulder pain reported positive results. One study evaluating pain and the sensation of heaviness in the affected limb during physical activities reported conflicting results. Two studies evaluating BCRL reported positive results, three studies reported no significant results, and one study reported conflicting results. One study evaluating the thickness of the subcutaneous tissue and muscle of the upper limb reported positive results. A study evaluating body composition reported that it did not yield any results. No major adverse events were reported in these studies. Only one study reported that delayed onset of muscle soreness (DOMS) occurred in the RT group [27], two participants developed adhesive capsulitis (frozen shoulder) with progressive immobilization during the intervention programs in another study (The restrictions group was told to restrict the activity of the affected limb for 6 months) [31]. The patients were told to avoid heavy or strenuous PA, including aerobic or other types of exercise classes with heavy upper-limb PA or work, and to avoid carrying or lifting groceries or other items weighing more than 3 kg. One patient developed supraspinatus tendinopathy. One of these women probably had a latent frozen shoulder before entering the study [31].

### Lower extremity resistance exercises

Three RCTs [35-37] have applied lower extremity RT in women after breast cancer. Included 3 studies used leg extension, leg curl, 45° leg press, and calf raise [35, 36], one study used leg press [37].

Four studies evaluating maximum strength (leg extension, leg curl, 45° leg press, and calf raise) reported positive results. Two studies evaluating muscle power (45° leg press) reported positive results. One study evaluating quality of life has reported conflicting results. One study evaluating body composition reported positive results, while another study reported that no results could be obtained and one study reported conflicting results. Three studies evaluating self-reported fatigue reported positive results. One study evaluating muscle fatigue indicators and the assessment of maximal voluntary isometric contraction (critical torque [CT] and W prime [W’]) reported positive results. One study evaluating functional tests (The five times and 30-s sit-to-stand tests, chair stand (sec) and TUG) has reported conflicting results. One study evaluating the walking test reported positive results, while another study reported conflicting results. One study evaluating physical activity level (IPAQ) reported positive results. One study evaluating 6MWT reported positive results. One study evaluating circulating inflammatory markers has reported conflicting results. No major adverse events were reported in the studies.

### Combined upper and lower extremity resistance exercises

Five RCTs [38-42] have applied combined upper and lower extremity RT in women after breast cancer. One of the included studies used resistance exercises consisting of 5 upper body and 5 lower body exercises designed to utilize major muscle groups and employ functional movements (e.g. chair stands, lunges in multiple directions, calf raises, one-arm row, chest press, front/lateral shoulder raise, and push-ups) [38]. Another study used resistance machines and free weights (targeting muscles of the chest, back, shoulders, and arms, as well as the buttocks, hips, and thighs) [39]. Two studies used upper body exercises (seated row, supine dumbbell press, lateral or front raises, bicep curls, and triceps pushdowns) performed with dumbbells or variable resistance machines. Lower body exercises (leg press, back extension, leg extension, and leg curl) were performed with variable resistance machines [40, 41] and one study used high-intensity circuit RT [42].

Four studies reported improvements in maximum muscle strength (1 RM-Bench Press). One study evaluating muscle strength (1 RM- Chest Press) reported positive results. Three studies evaluating muscle strength (1 RM- Leg Press) reported positive results. One study evaluating maximum strength (leg extension, leg curl, 45° leg press, and calf raise) reported positive results. One study evaluating back strength reported no change. One study evaluating muscle power (45° leg press) reported no change. One study evaluating handgrip strength reported positive results, while another reported that no change could be obtained. One study evaluating quality of life reported no change. One study evaluating BCRL reported positive results, while another study reported that no change could be obtained. One study evaluating body composition reported positive results, while another study reported that no change could be obtained, and another study reported conflicting results. Two studies evaluating function tests (Five times and 30-s sit-to-stand tests, Chair Stand (sec), and TUG) reported positive results. Two studies evaluating 6MWT reported positive results. One study evaluating circulating inflammatory markers reported no change. One study evaluating physical function, including SF-36, Short Physical Performance Battery (SPPB), and Late Life Function and Disability Instruments, reported positive results, while one study reported conflicting results. One study evaluating flexibility (Chair Sit and Reach (cm)) reported positive results, while another study reported that no change was observed. One study evaluating walk speed (4m walk) reported positive results. One study evaluating body size reported conflicting results. Two studies evaluating biomarkers reported conflicting results. One study evaluating body image reported positive results. One study evaluating muscle endurance reported that no change was observed. One study evaluating reaction time reported positive results. One study evaluating balance reported conflicting results. Only one participant experienced a study related injury (wrist injury) that prevented continued participation [39].

## Discussion

To the best of our knowledge, this is the first systematic review to evaluate and compare the effects of RT performed for the upper extremities alone or for the lower extremities alone or combined upper and lower extremities on the outcome measurements in patients with breast cancer. The main findings of this systematic review indicate that upper extremity RT programs reduce shoulder pain, and arm activity limitation and improve the thickness of subcutaneous tissue and muscle strength of the upper limbs. The effects of upper extremity RT on body composition, pulmonary function, BCRL, pain and the sensation of heaviness in the affected limb during PA, quality of life, hand-grip strength, and shoulder ROM are still unclear. Lower extremity RT programs improve lower body maximum strength, muscle power, level of PA, functional exercise capacity, and decrease muscle fatigue. The effects of lower extremity RT programs on quality of life, body composition, walking and functional capacities, level of PA, and inflammation are still unclear. Upper and lower extremity combined RT improves functional exercise capacity, muscle strength, walking speed, muscular endurance, body image and reaction time. The effects of combined RT on hand-grip strength, quality of life, BCRL, body composition, inflammation, physical function, flexibility biomarkers, balance, muscle strength and back endurance are still unclear.

It is known that approximately 20% of patients with breast cancer develop BCRL due to the treatments applied [43]. As it is known, BCRL is associated with many negative symptoms such as pain, feeling of heaviness, tension, decreased ROM, impaired fine motor skills, restriction in ADLs, and decreased quality of life [43]. Until the early 2000s, patients with breast cancer were advised to avoid vigorous, repetitive upper extremity exercises that could cause the development of lymphedema or increase the existing lymphedema [40]. However, studies have shown that resistance exercises do not cause the formation of lymphedema or increase the existing lymphedema, and they provide many benefits, such as maintaining and regaining the physical function of the affected arm and achieving a healthy body composition [43]. It has been reported that the RT program in patients with breast cancer is safe and will not cause any worsening of lymphedema symptoms or severity when the program and protocol are customized according to the patient, and the changes that may occur during the training are closely monitored [44, 45]. In our study, we observed that only upper extremity RT and combined upper and lower extremity RT had positive effects on BCRL. However, there were contradictory statements in some studies related to this parameter. Therefore, it is necessary to conduct studies of higher quality on this parameter in future research.

Treatment modalities such as chemotherapy, radiotherapy, endocrine therapy and immunotherapy applied during breast cancer treatment cause a decrease in muscle strength and cardiovascular fitness, adverse changes in body composition such as loss of lean mass, increase in fat mass, and decrease in quality of life with cancer-related fatigue, anxiety, depression and pain. In addition, changes in body composition are associated with breast cancer-related mortality and even have critical effects on metabolism, inflammation, and the immune system of skeletal muscle and adipose tissue [46]. Studies have reported that exercise is safe and effective during cancer treatments, reduces recurrence by 40%, reduces body weight and fat mass in the long term, and increases lean mass when combined with dietary intervention. All these changes don’t only affect body composition but also contribute to physical and psychological health. In patients with breast cancer, a structured exercise training program improves muscle strength and cardiovascular fitness, as well as cancer-related fatigue, anxiety, depression and pain, leading to improved quality of life [46]. A 12-week upper and lower extremity combined RT program in patients with breast cancer resulted in a decrease in fat mass, an increase in lean body weight, an increase in muscle strength in both upper and lower extremities, an improvement in quality of life, and an improvement in cardiovascular fitness [46]. In our study, RT targeting the upper extremities alone, lower extremities alone, or both combined resulted in increased muscle strength, improved body composition, and enhanced quality of life. However, the certainty of this evidence is very low due to methodological limitations, inconsistencies across studies, and imprecision arising from small sample sizes. Consequently, it is not possible to determine the most effective type of training based on the available data.

This systematic review demonstrates that RT has a positive effect in breast cancer patients and that the effects caused by the type of RT administered differ. RT can be administered at various stages of breast cancer treatment. Studies have reported that RT does not exacerbate lymphedema symptoms, adverse events related to the musculoskeletal system are rarely seen, it may affect quality of life, and it may positively affect aerobic exercise capacity depending on the RT program administered [18, 45] After RT, it has been reported to positively impact joint ROM and muscle strength, especially in the post-breast cancer surgery period. Any adverse effects were also reported in the studies [19]. However, more studies are needed on how several types of RT will impact muscle strength and ROM.

## Strengths and limitations

A key strength of this systematic review is a rigorous methodology, including an extensive search to assess the certainty of evidence, which was not performed in similar reviews [19, 43]. However, this review has some limitations. Since persistent symptoms after breast cancer is a relatively new condition, the majority of the included studies have a short follow-up period, which impacts the certainty of evidence. The limited number of RT studies conducted in individuals with breast cancer has led us to consider studies performed with weight and elastic equipment. In addition, the range of outcomes for the same intervention, as well as intervention heterogeneity, might limit the generalizability of our findings. We could not summarize the results quantitatively. Moreover, this review did not consider the effect of the interventions on some other important symptoms that are commonly reported by these patients, such as general pain or discomfort, impaired upper extremity, joint pain, and fatigue, and with heterogeneity in the number and severity of symptoms experienced [6]. Finally, few studies included in this review reported on the adverse events with the therapies applied. There are reports of back and wrist pain after RT, which may require a reduction or even cessation of the training program. In this context, more research is needed on the place of RT interventions.

## Implications for clinical practice and further research

These findings can guide physiotherapists in treating patients with breast cancer. Certainty of evidence of included studies is very low (PEDro score between 4 and 9) due to methodological weaknesses, heterogeneity of outcomes and training parameters, and small sample size. Given the paucity of evidence, high-quality and rigorous studies are needed to confirm our hypotheses. Future studies should preferably have a controlled design and should include a sufficient number of participants. Adverse events of RT programs in breast cancer must be studied further. Furthermore, the evidence is particularly scarce for the following intervention effects: the effects of combined upper and lower limb RT intervention on lymphedema, functional capacity, and body composition.

## Conclusions

RT programs may be considered in the rehabilitation program after breast cancer was performed as a stand-alone intervention in the upper extremity, in the lower extremity, or in the combined upper and lower extremities. These exercises improve upper and lower extremity muscle strength and physical function. However, the certainty of the evidence is very low, indicating significant uncertainty about these effects. Further studies should focus on the modification of RT to be applied after breast cancer according to the needs of the patients and should be planned to provide maximum benefit and randomized controlled studies consisting of larger study groups in which stand-alone intervention upper extremity, lower extremity or combined upper and lower extremity resistance exercises are needed. Additionally, future studies are needed to investigate the effects of combined upper and lower extremity exercise training on BCRL, muscle strength, upper extremity functionality, and body composition.

## Data Availability

The data that support the findings of this study are available from the corresponding author, [HO], upon reasonable request
